# Abnormal-Sound Diagnosis for Kaplan Hydroelectric Generating Units Based on Continuous Wavelet Transform and Transfer Learning

**DOI:** 10.3390/s24237441

**Published:** 2024-11-21

**Authors:** Yu Liu, Zhuofei Xu, Pengcheng Guo, Longgang Sun

**Affiliations:** 1 School of Water Resources and Hydroelectric Engineering, Xi’an University of Technology, Xi’an 710048, China; liu.wenyu5@163.com (Y.L.); sunlg@xaut.edu.cn (L.S.); 2 Faculty of Printing Packaging Engineering and Digital Media Technology, Xi’an University of Technology, Xi’an 710048, China

**Keywords:** abnormal-sound diagnosis, Kaplan hydroelectric generating units, transfer learning, acoustic signal recognition

## Abstract

To realize abnormal-sound diagnosis in hydroelectric generating units, this study proposes a method based on continuous wavelet transform (CWT) and Transfer Learning (TL). A denoising algorithm utilizing spectral noise-gate technology is proposed to enhance fault characteristics in hydroelectric units. Subsequently, Continuous Wavelet Transform is applied to obtain frequency components, and the results are converted into a series of pseudo-color images to highlight information differences. A transfer model is subsequently developed for feature extraction, utilizing simplified fully connected layers to reduce modeling costs. The study optimizes key parameters during the signal-processing stage and achieves an improved parameter-setting scheme. Acoustic signals corresponding to four different fault states and a normal state are collected from a Kaplan hydroelectric generating unit in a hydropower station. The signal diagnosis accuracy rates before filtering are 84.83% and 95.14%. These rates significantly improved to 98.88% and 98.06%, respectively, demonstrating the effectiveness of the noise-reduction process. To demonstrate the superiority of the improved model in this work, a series of classic deep-learning models, including AlexNet, Resnet18, and MobileNetV3, are used for comparative analysis. The proposed method can effectively diagnose faults in Kaplan hydroelectric generating units with a high accuracy, which is crucial for the daily monitoring and maintenance of these units.

## 1. Introduction

Among various hydroelectric machines, Kaplan hydroelectric generating units are suitable for a range of medium-to-low water heads and are widely used. Besides playing a crucial role in the supply of clean and renewable energy in the power grid, they have also increasingly taken on the function of power adjustment in recent years. Their operating conditions are facing more stringent running requirements with rapid load changes and responses. It is essential to conduct condition monitoring and fault diagnosis to ensure the long-term safe and stable operation of these units [1,2,3].

Recently, with the rapid advancements in testing technology and artificial intelligence, data-driven condition-monitoring and fault-diagnosis methods utilizing machine learning have been applied to various hydroelectric units [4,5,6]. In these methods, monitoring information obtained by sensing technology is used to establish recognition models to realize monitory and fault diagnosis. Vibration and swing signals are most commonly chosen due to their higher reliability and real-time performance. However, vibration and swing signals are always restrained by the requirement for contact measurement and are difficult to obtain in many critical areas. As such, more research has started to focus on acoustic signals in fault monitoring and diagnosis [7,8]. Abnormal sounds in mechanical equipment always contain a wealth of information, such as frequency, amplitude, harmonics, and other characteristics, that can reflect various internal flow abnormalities, mechanical faults, and electromagnetic faults in a hydroelectric unit with non-contact measurement [9,10,11]. Therefore, many mechanical equipment faults can be identified through abnormal sounds during operation, and research on abnormal-sound diagnosis holds significant engineering application value.

Compared with vibration or swing, acoustic signals always contain heavy noise and experience significant frequency loss when passing through water and air. For hydroelectric units, the phenomenon of strong noise is much more pronounced. Under the top cover of the units, one can hear nothing but a very loud roar. The entire audible frequency range is disturbed, making acoustic signals hard to distinguish [12,13]. The filtering effect is crucial for subsequent fault diagnosis work since a typical fault is always related to a specific frequency value. To realize acoustic signal denoising, a signal filtering method is always employed. B. M. R. Bharathi et al. [14] developed an Empirical Mode Decomposition (EMD) maximum-likelihood time-delay estimation method for low-frequency and low-SNR acoustic signals generated by underwater machinery in a reverberant environment. Shuyao Liu et al. [15] collected cutting force and acoustic emission signals, analyzed the effect of shear and plowing effects on the frequency of acoustic emission (AE) signals in machining, and built a relationship between the components using VMD and the shearing and plowing process, respectively. It should be noted that both EMD and VMD are limited when facing a complex frequency property or non-stationary signal. The high computational complexity required and the difficulty in controlling parameters are the key issues that constrain their application [16]. Fang Dao et al. [17] proposed wavelet transform (WT) and Ensemble EMD methods for denoising the acoustic signals of hydro-turbine runners under normal and sand-laden water flow conditions, which serves as a valuable supplement to the existing hydro-turbine condition-monitoring and fault-diagnosis system mostly based on vibration, and WT was effective in preserving multiple-scale information [18,19]. Yuan Xie et al. [20] proposed an adaptive generalized network that learns the characteristics of underwater sound at different frequencies by converting fixed wavelet parameters into fine-grained learnable parameters, and its flexible and fine-grained design is conducive to capturing more background acoustic information.

Another approach to heavy acoustic noise reduction is spectral gating, a spectral subtraction algorithm that is characterized by strong real-time performance and independence from prior models [21]. Sainburg et al. [22] and Stowell et al. [23] effectively applied stationary and non-stationary spectral gating noise reduction to common birdsong with airplane noise in the background. Taking into account computational complexity and the characteristics of hydroelectric unit noise, an analysis method is proposed in this work based on spectral gating and WT. First, improved spectral gating is used to remove background sounds, and then a filtered acoustic signal is analyzed using the WT method to obtain spectral information on faults. Since the signal sequence has a high sampling frequency, Continuous Wavelet Transform (CWT) is chosen, which allows continuous adjustment between scales, providing a more flexible resolution.

Regarding abnormal-sound diagnosis models, these comprise mainly shallow neural network models and deep-learning models. Tauheed Mian et al. [24] presented a methodology involving the detection of bearing faults using sound quality metrics based on a Support Vector Machine (SVM). Y. S. Wang et al. [25] proposed a preprocessing method for noise-based engine-fault diagnosis based on an artificial neural network (ANN). Xiaoyuan Zhang et al. [26] established a novel classifier combining rough sets and SVM for hydroelectric generator units. However, hydroelectric units are always highly integrated, large-scale plants, and it is inadequate for dealing with complex fault modes by solely relying on these models. Limited-sample and imbalance problems are also drawbacks for shallow models. The deep-learning model has strong abilities in multi-level feature extraction, and it can extract high-level features and mine complex relationships from fault data. Convolutional Neural Networks (CNNs) are among the most commonly used models in fault diagnosis. Zidong Yu et al. [27] proposed a time-scale adaptive CNN model that achieves better performance under both noise interference and complex working conditions for rotating machinery. Jisoo Kim et al. [28] developed an operation monitoring system by analyzing sound, and the system could detect the operation states based on the log-Mel spectrogram and CNN. Still, regarding deep networks, computational resource demand is always an unavoidable problem. To improve the efficiency of CNN, Depth-wise Separable Convolution Neural Network (DSCNN) and Transfer-Learning (TL) methods are employed. DSCNN consists of depth-wise convolution and pointwise convolution to reduce dimensionality [29,30]. Muhammad Irfan et al. [31] proposed a novel separable convolution-based autoencoder network for the classification of underwater acoustic datasets. Zhuofei Xu et al. [32] developed a Siamese network composed of two DSCNN sub-models in rotating machinery fault diagnosis. DSCNN can reduce parameter numbers and decrease model-training costs. Now, CNNs and DSCNNs possess sufficient recognition capabilities to handle complex diagnosis tasks, but they are not particularly adept at addressing issues related to the number of fault samples and sample imbalance. Therefore, we are directing our focus towards TL models.

TL refers to a machine-learning technique in which a model trained on one task is adapted for a second related task. In the context of deep learning, transfer learning involves using a pre-trained neural network model as a starting point and fine-tuning it for a new task. This approach is particularly useful when there is limited data available for the target task, as it leverages the knowledge and features extracted from a large dataset used for pre-training. Jie Liu et al. [33] proposed a spatiotemporal CNN model to address the issue of inconsistent accuracy under different operating parameters in nuclear power fault diagnosis, and the model demonstrates improved speed for cross-operating condition-fault diagnosis by employing a transfer-learning strategy. Pengqian Liu et al. [34] proposed a CNN-TL model for pipeline leakage detection under multiple working conditions by acoustic emission signals, and the CNN-TL model requires less computational power and can accomplish accurate detection. Since the TL-CNN model does not require a parameter-training process, it possesses strong robustness and adaptability. Moreover, the TL method can also play a good role in small-size or unbalanced sample data. As large-scale integrated equipment, each hydroelectric generating unit has its unique work environment and geographical location, causing acoustic signals to vary with the environment even for the same model of equipment under the same operating conditions. Thus, a trained diagnosis model for a certain unit can only be used once, and a new model needs to be recreated when facing another unit of the same model or even after a repair. Considering the factors of training cost, acoustic diagnosis performance, and sound properties in hydroelectric units, a TL method is more suitable in this context.

Therefore, this paper proposes a method to improve the diagnosis of abnormal sounds in hydroelectric units. Acoustic signals are first converted and then analyzed using a transfer-learning model. Compared to similar existing studies, the model developed in this research primarily derives its information from augmented time-frequency images, effectively simplifying the model-building process. The novelties and contributions of this work are summarized as follows:

(1) According to the acoustic properties inside the hydroelectric unit, an improved acoustic spectrum subtraction method is proposed based on spectral subtraction and noise gating. It can effectively eliminate the low-frequency band information and suppress environmental noise to highlight fault characteristics.
(2) A method for visualizing acoustic signals is proposed using continuous wavelet transform. Wavelet coefficients of signals are obtained first and then transformed into pseudo-color digital images to represent time-frequency information. Specific regions of digital images are utilized to highlight the high-frequency features typically present in abnormal acoustic signals.
(3) A transfer-learning model has been developed for abnormal-sound diagnosis, achieving high accuracy and good performance. The feature-extraction process is simplified, and the decision module is specifically designed for diagnosing abnormal sounds in hydroelectric units, significantly reducing the number of parameters required for training the model.

## 2. Diagnosis Algorithm for Acoustic Signals

### 2.1. Acoustic Denoising Algorithm

In the working environment of hydroelectric units, strong noise pervades the entire space under head cover. People even cannot hear shouts from each other at a distance of about 10 cm to 20 cm. This noise originates not only from strong water flow but also continues impacts between water, guide vane, and turbine blade. Water impaction noise in a turbine always contains a very strong power but a low frequency since high-frequency sounds are easily absorbed in water while low-frequency sounds tend to travel further. Consequently, acoustic signals in water often exhibit lower frequencies since high-frequency sounds attenuate during propagation. To overcome strong noise in acoustic recognition, spectral subtraction is selected based on the property of acoustic signals from the water turbine. Spectral subtraction is a technique used in audio signal processing, particularly in the field of noise reduction and speech enhancement. Its primary purpose is to reduce or eliminate unwanted noise from an audio signal while preserving the quality of the desired signal. Spectral subtraction is suitable for relatively stable background noise like water flow here.

For a hydroelectric generating unit, it must be noted that sound signals change with collection positions even under the same operation state. Therefore, a series of background signals should be recorded and used correspondingly according to the collection position. Considering that when faults or abnormal states occur in mechanical equipment, new frequency components always appear compared to signals under normal running states. Thus, we take signals under normal running state as background noise here. If there are no faults, the frequency will be the same as the background noise. Monitoring signals can be filtered using the spectral subtraction method and then analyzed for further analysis. After denoising, fault information can be identified through frequency components.

**Figure 1:**
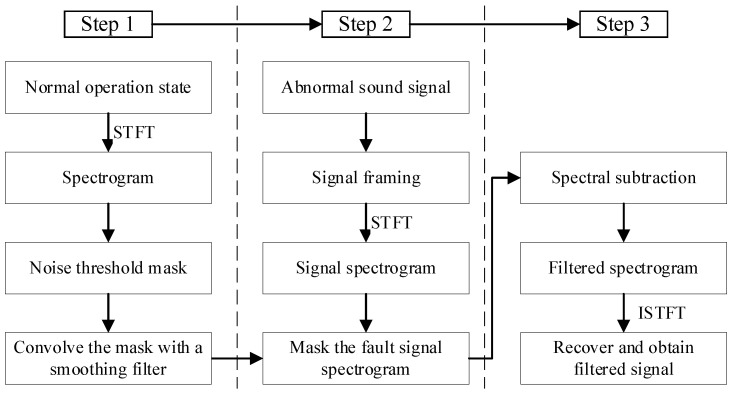
Acoustic signal denoising algorithm for hydroelectric units.

According to the description above, an improved spectral subtraction method for units is proposed based on spectral gating and Short-Time Fourier Transform (STFT), as shown in Figure 1. The calculation steps are as follows:

Step 1: the spectrogram of signals under normal operation state is calculated over the noise audio clip based on STFT by Formula (1).

(1) $$F(t,\omega )=\int_{-\infty }^{\infty }f(\tau )w(\tau -t)e^{-i\omega \tau }d\tau$$

where F(t,ω) means STFT at t time and frequency ω. f(τ) is the input signal. w(τ−t) is the window function, typically a function localized in the time domain. $e^{-i\omega \tau }$ is the complex exponential representing frequency ω. In the discrete case, the integral becomes a sum, and the calculation is performed using the inverse transform of the Fourier transform. The purpose of the STFT is to provide a local representation of the signal in both time and frequency, allowing for a better understanding of the signal characteristics at different time and frequency points.

Step 2: Signals obtained when hydroelectric working under normal operation states are considered to be background noise, and we assume that the fault signal is a fusion result of background noise and more abnormal high-frequency components. Then, take the obtained noise STFT spectrogram as a threshold matrix, which is known as a mask here, and apply a smoothing filter to the mask to prevent frequency leakage and distortion issues. Second, calculate the STFT spectrogram over the signal to be diagnosed after framing it to the appropriate size, and then make a comparison between mask value and spectrogram value.

Step 3: Subtract the mask value from the spectrogram value, and the result is a filtered spectrogram. Then, recover the time sequence from the spectrogram using the inverse transform of STFT, as shown in Formula (2)

(2) $$f^{\prime }(t)=\int_{-\infty }^{\infty }F(\tau ,\omega )\cdot w(\tau -t)\cdot e^{j\omega \tau }d\omega$$

F(τ,ω) represents the local characteristics of the signal at τ time and frequency ω obtained from STFT. $f^{\prime }(t)$ is the reconstructed filtered signal after the inverse transform.

### 2.2. Acoustic Signal Analysis and Visualization

#### 2.2.1. Continuous Wavelet Transform Analysis

Continuous wavelet transform (CWT) is utilized here to extract fault information of units from filtered sound signals, and the resulting time-frequency spectra can be used for modeling and training of diagnosis models. Acoustic signals can be analyzed at different scales by basic wavelet functions, and a series of wavelet coefficients will be obtained as analysis results. CWT is defined as Formula (3).

(3) $$W_{f}\left(a,\tau \right)=\frac{1}{\sqrt{a}}\int_{-\infty }^{+\infty }f^{\prime }(t)\psi *(\frac{t-\tau }{a})dx$$

where ∗ means a complex conjugate of mother wavelet function ψ(⋅). Morlet wavelet basis is chosen here because it has a constant peak value in both time and frequency domains, which gives it excellent localization properties for wavelet transforms. It closely resembles the attenuated components of impact responses commonly found in various mechanical faults [35,36]. Morlet wavelet basis can be expressed as Formula (4)

(4) $$\psi_{morlet}(t)=e^{i\omega_{0}t}e^{-\frac{t^{2}}{2}}$$

Morlet wavelet is composed of a complex trigonometric function multiplied by an exponential decay function and $\omega_{0}$ means center frequency here.

#### 2.2.2. Visualization Based on Digital Image Processing

Wavelet coefficients can be expressed as a matrix, where the columns and rows reflect frequency information at different positions and scales. Regarding a deep-learning model for diagnosis, it is also necessary to reduce both the size and number of coefficients to save computational resources and improve computational efficiency. To identify the time-frequency information hidden in the wavelet coefficients matrix, the results of wavelet transform are often converted into digital image format so that they can be recognized by CNN models or other advanced networks.

The conversion process for digital images is as follows. Values in matrix coefficients are first normalized to a range between 0 and 255 as per Formula (5). Normalization is a preprocess for data visualization and $\left[\cdot \right]$ means integer arithmetic here.

(5) $$x=\left[\frac{\left|x_{\max}-x^{\prime }\right|}{x_{\max}-x_{\min}}\times 255\right]$$

where $x^{\prime }$ and x represents input value and normalized one, respectively. Then resize the normalized matrix into a square matrix to fulfill the shape of the image needed in modeling.

The CNN model can simultaneously learn features from different color channels, allowing it to be more flexible and powerful when dealing with various colors and textures separately. To make the recognition model understand acoustic information better, images are typically input in RGB three-channel form. We can obtain a gray image from Formula (6) and then change it into pseudo-color RGB images.

**Figure 2:**
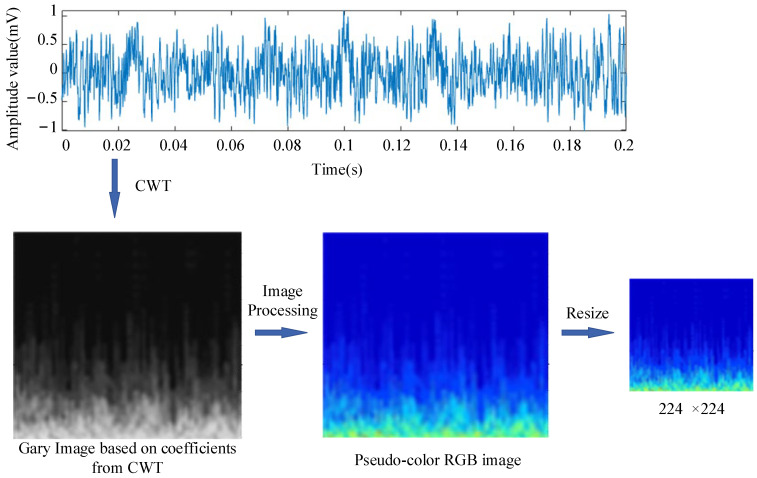
Images obtained from CWT.

After format conversion for analysis results, as shown in Figure 2, acoustic signals from hydroelectric units are converted into a series of images containing multiple-scale time-frequency information. These digital images are used as input for the diagnosis model.

### 2.3. Parameter Selection for STFT and CWT

#### 2.3.1. Parameters in STFT

In the process of STFT, parameters are selected based on signal characteristics. The window length determines the frequency resolution of each time slice. For stationary signals or scenarios requiring detailed frequency information, a longer window should be used. For rapidly changing signals or when capturing transient events is necessary, a shorter window is more appropriate. Since many abnormal phenomena in hydropower units associated with sounds tend to persist over time, there is a tendency to moderately increase the window length.

**Figure 3:**
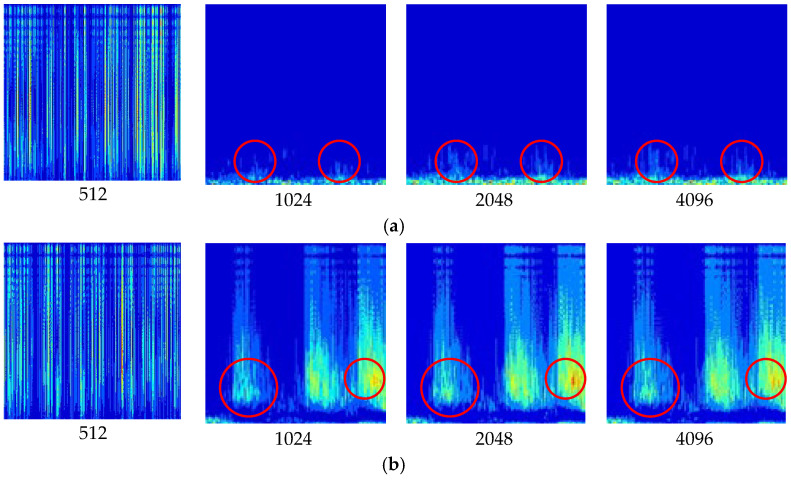
CWT images with different window lengths: (**a**) Normal running sound in the hydroelectric unit; (**b**) Metal collision in the hydroelectric unit.

Filtered signals with a window length between 512 and 4096 are shown in Figure 3, and significantly changing areas are marked with red circles for comparison. When the length is below 1024, it cannot provide an effective analysis due to the very low-frequency resolution. Compared with the results between 1024 and 2048, the images with 2048 have more detailed components, which can be clearly found by observing the part within the red circles. Increasing the window length from 2048 to 4096 reveals no obvious new spectral information, and this indicates that increasing the window length has little significance. Therefore, we set 2048 as the balancing computational efficiency and frequency resolution. The step size determines the overlap between consecutive windows, specifically the number of audio samples the window moves over each time. Considering the high sampling precision of hydroelectric unit signals in this study, a smaller step of 25% window size was chosen to enhance time resolution.

#### 2.3.2. Parameters in CWT

**Figure 4:**
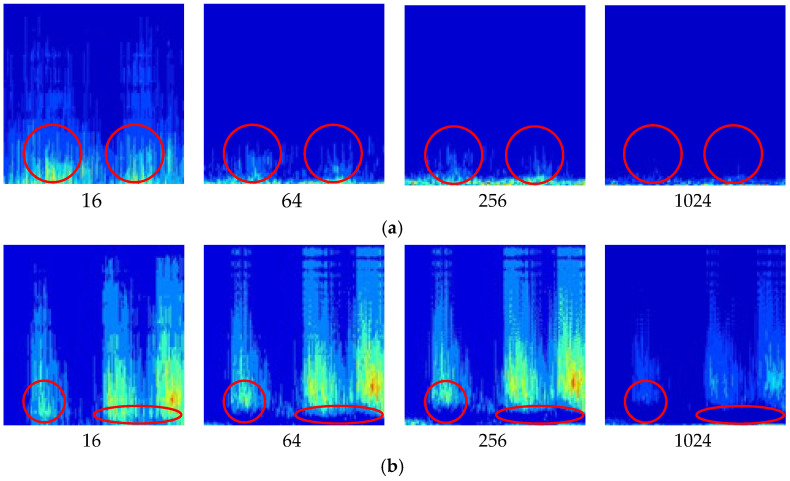
CWT images with scale factors: (**a**) Normal running sound in the hydroelectric unit; (**b**) Metal collision in the hydroelectric unit.

For abnormal-sound diagnosis in Kaplan hydropower units, acoustic signals typically contain frequency information from various mechanical vibrations and rotating components. The Morlet wavelet can provide a more detailed frequency analysis, which helps identify and diagnose complex mechanical issues. After determining the wavelet function, the scale factor is the key variable in determining the time-frequency image. The study conducted a comparative analysis of the signal within a series of scale factors ranging from 16 to 1024 and recorded the results in Figure 4. Significantly changing areas are marked with red circles.

By comparing the areas marked with circles in Figure 4a, it can be observed that the frequency components in Figure 4a(16) suddenly increase significantly. This is due to the excessively low-frequency resolution, which has greatly stretched the pixels in the vertical direction, resulting in severe distortion. A similar situation also occurs in Figure 4b, where the low-frequency components in the areas marked in red have almost completely disappeared in Figure 4b(16), replaced by green pixels. Therefore, a small scale factor of 16 provides higher time resolution but may fail to capture low-frequency information, leading to image distortion. In contrast, large scale factors of 1024 offer higher frequency resolution but reduce time resolution, resulting in the loss of transient changes and detailed features of the signal. In Figure 4a(1024) and 4b(1024), almost all frequency components have disappeared, rendering the image meaningless. Within the range of 64 to 256, there are no significant differences in the images. To balance both time and frequency characteristics, a scale factor of 256 was selected as optimal.

## 3. Diagnosis Model for Abnormal Sound

### 3.1. VGG16 Model for Transform Learning

The development of fault diagnosis in hydroelectric generating units is always restricted by limited fault data. Once there is an obvious fault in a unit, the entire unit and even regional power grids will face huge dangers, which is not acceptable in actual work. Consequently, oversaturated maintenance work is always undertaken for units, and a few obvious faults data can be collected. Therefore, the transfer-learning method is chosen to address these problems. Among common TL models, the VGG16 network is a pre-trained model that can be directly used for feature extraction and recognition of images. Because the VGG16 model has already been trained on the ImageNet database, which contains over 14 million annotated images across more than 21,000 categories, with over 1 million images that have precise bounding box annotations, it has a strong capability for image-pattern recognition and requires little fault data, making it very suitable for our work. Thus, we construct an improved abnormal-sound diagnosis model based on the VGG16 core.

**Figure 5:**
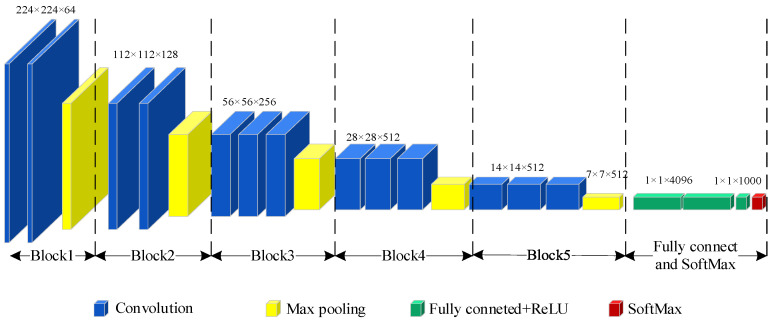
The network structure of VGG16.

The VGG-16 network structure is shown in Figure 5. It consists of 16 layers, which include 13 convolutional layers and three fully connected layers. It also incorporates five max-pooling layers and a SoftMax layer to output the final result. The VGG-16 structure is divided into five blocks: Blocks I and II have two convolutional layers and one max-pooling layer to extract low-level features from time-frequency images. Blocks III, IV, and V consist of three convolutional layers and a max-pooling layer to extract deep-level features. Each block utilizes 3 × 3 convolutional kernels and is followed by 2 × 2 max-pooling layers, with strides set to 1 and 2 in convolution and pooling, respectively. The first convolutional layer has 64 channels, while the last one has 512 channels through a series of max-pooling calculations.

Five blocks are used to obtain features. Fully connected layers and the SoftMax layer are used for classification. ReLU activation function is chosen in fully connected layers to enhance the capability of non-linear transformations in the model as Formula (6).

(6) ReLU(x)=max(0,x)=x,x>00,x≤0

Due to its simplicity, the ReLU function offers fast computation speed and quicker model convergence. Its zero output for negative values increases sparsity in VGG16, which can reduce overfitting effectively.

### 3.2. Diagnosis Model Based on VGG-16

**Figure 6:**
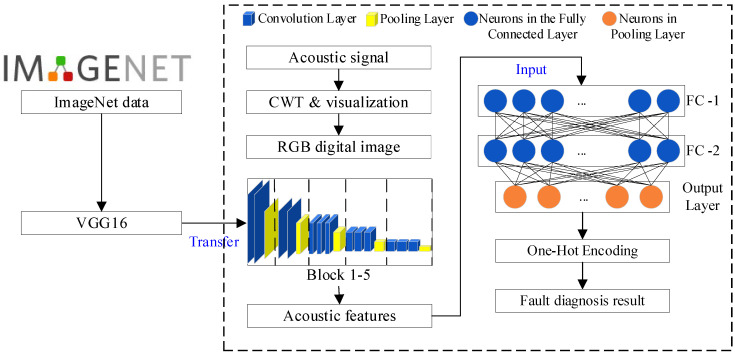
Improved VGG16 model.

The VGG-16 model demonstrates better performance in image-feature extraction and learning ability due to the added convolutional layers. However, the large number of parameters also leads to an increase in training time. Meanwhile, an effective mechanism to prevent gradient vanishing is needed to avoid slow convergence and gradient explosion during training. An improved model, as shown in Figure 6, has been built to address these issues. Five blocks from VGG-16 are retained, and a signal-visualization module is used as the input layer. Then, a highly efficient classification section has been restructured to reduce the model-training time and suppress overfitting.

Regarding fault diagnosis of hydroelectric units, fault classes are much fewer compared to the ImageNet database, which has nearly 15 million pictures and 22,000 classes. Three fully connected layers are not necessary, so they are removed to decrease the parameter number and improve running efficiency. Keep five blocks to extract features, and two fully connected layers are added to connect Block 5 and the output layer, which are noted as FC-1 and FC-2 in Figure 6. An output layer is also added with SoftMax activation function as Formula (7)

(7) $$Soft\max(i)=\frac{e^{z_{i}}}{\sum_{j=1}^{n}e^{z_{j}}}$$

where $z_{i}$ means a score for i−th category and n represent the whole number of faults to be identified. The output of the SoftMax function is the probability for i−th category. With the improved model, most of the already trained VGG-16 can be used, while the fully connected and output layers are replaced with much simpler ones. In the improved model, only the parameters in FC-1, FC-2, and the output layer need to be trained. This significantly solves the issues of slow convergence and gradient explosion.

Categorical cross-entropy is chosen as the loss function for multiclass faults classification as Formula (8). The formula for categorical cross-entropy is as follows, where $p_{i}(x)$ is the predicted probability distribution for x and $q_{i}(x)$ is the true class label, and k is class number.

(8) $$Categorical\;Cross-Entropy=-\sum_{i=1}^{k}q_{i}(x)\log[p_{i}(x)]$$

One-hot encoding is used to fulfill the loss function as the output format since it simplifies the representation of categorical output in diagnosis tasks, making it compatible with various TL algorithms, loss functions, and evaluation metrics.

## 4. Experiment Verification

### 4.1. Application Target and Data Description

**Figure 7:**
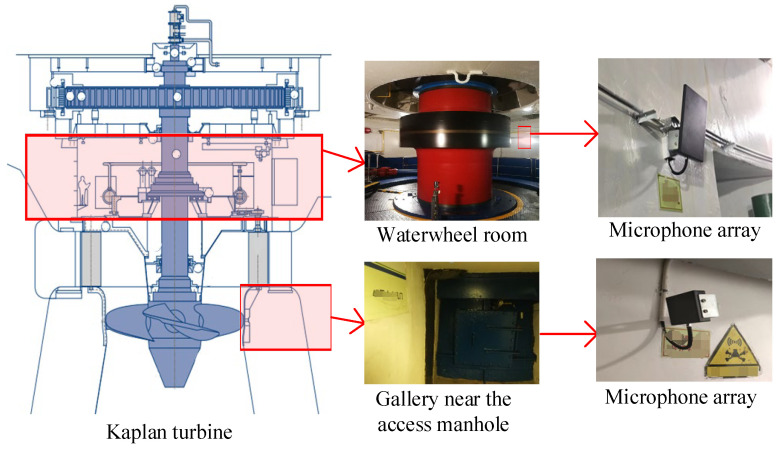
High-frequency microphone array.

This paper adopts a Kaplan hydroelectric generating unit in a hydropower station located in Southwest China, as shown in Figure 7. An acoustic signal acquisition system has been designed and deployed in some key areas of units, mainly including the waterwheel room and gallery near the access manhole. The acoustic monitoring system contains a high-frequency microphone array. Considering that mechanical fault information is often hidden in the high-frequency range, microphones with a high sampling frequency of 96 kHz have been used. Acoustic signals can be obtained, which are already large beyond the range of human auditory perception.

For an in-service unit, the occurrence of significant faults during daily operations is unacceptable, as it can lead to severe disasters and huge safety risks, and simulated fault acoustic signals are used. To obtain various acoustic signals containing strong background noise of the running unit, abnormal and fault acoustic signals are obtained directly or simulated by audio equipment in the waterwheel room and gallery, and then fault sounds are collected and recorded by microphones in the waterwheel room and gallery.

All data can be categorized into two kinds: normal data and faulty data. Normal data noted as Class A represents the sound from the hydroelectric generating set in a normal running state near the rated power. Faulty data are abnormal, and faulty ones are noted as Class B to E. Class B simulates the sound of a buzzer when the electrical system alarms. Class C simulates the sound of metal rubbing, similar to the scratch sound when main shaft faults happen. Class D is a high-frequency wave signal, which always comes out as a high-frequency noise or an abnormal signal in various mechanical faults. Class E is a metal collision, always introduced by the loosening or falling-off of some metal parts.

Class A was obtained from the real working environment inside the waterwheel room and gallery. Class B and Class C were several segments of historical fault sound from the hydroelectric generating unit used in this work recorded between 2016 to 2021. Class D was used to simulate various mechanical faults, comprising frequencies ranging from 4 kHz to 20 kHz, and it was synthesized through code and converted into audio files. Class E was generated in the real environment by workers. They directly produced the corresponding collision sounds using bolts, nuts, and other components. The simulated data (Class B, C, and D) can be applied to real-world scenarios because the instruments we selected for collecting sound signal samples have a sampling frequency of 44.1 kHz, and the frequency response range of the playback equipment reaches 20 kHz, covering the main range of the original sound signals.

The sample sizes and classes are recorded in Table 1 and Table 2. Two groups of data were collected, including four fault states and one normal running state. All data are divided into training sets, validation sets, and test sets with a ratio of 3:1:1 for random training and evaluation. Each sample consists of a 1-s acoustic time sequence. Class B and C are limited because problems rarely occur in the hydroelectric generating units during actual operations. Class A, D, and E can be obtained or simulated with a large amount of data theoretically, but we may be restrained by experimental permits and power plant management regulations sometimes. Therefore, we encounter challenges related to small sample sizes and data imbalance to some degree.

### 4.2. Acoustic Signal Analysis

All acoustic signals are calculated according to the denoising algorithm and CWT method from Section 2.1 to Section 2.2 and then converted into RGB digital images with a size of 224 × 224 to analyze by deep-learning models.

**Figure 8:**
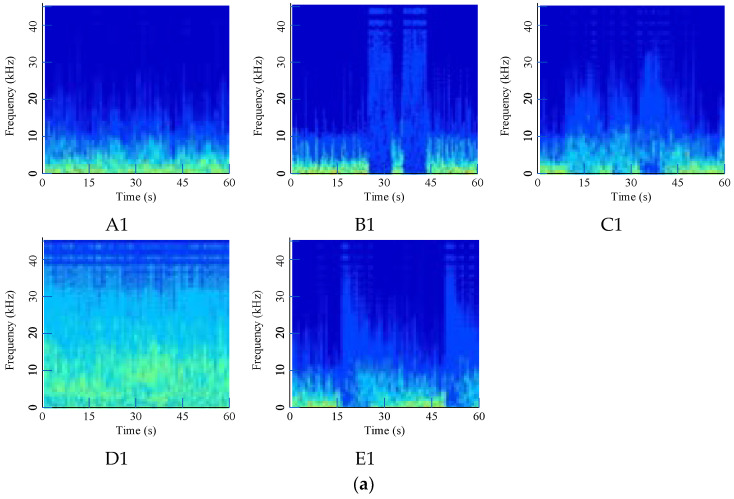

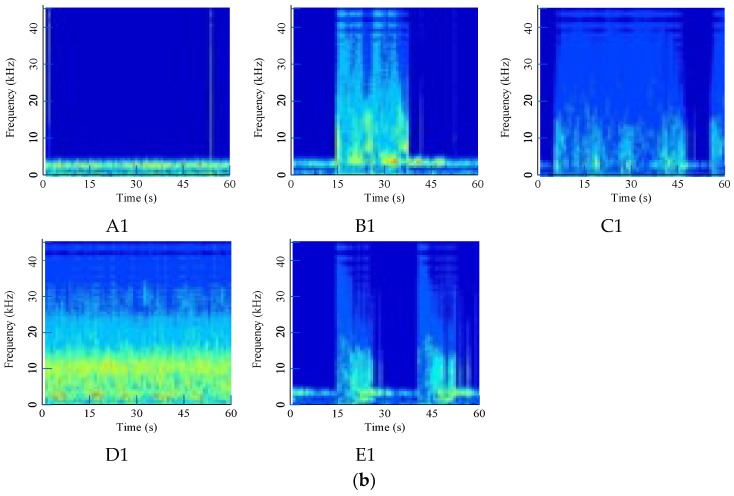
Time-frequency images of original acoustic signals: (**a**) Waterwheel room; (**b**) Gallery near the access manhole.

To demonstrate the effect of the denoising method, original signals are directly converted and shown in Figure 8. Time-frequency are severely fused, making them difficult to distinguish from each other, and the noise in the waterwheel room is much stronger due to the loud water sound.

**Figure 9:**
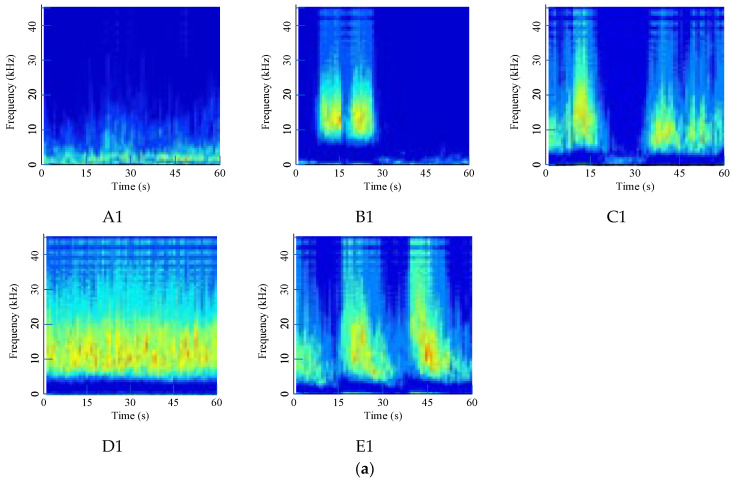

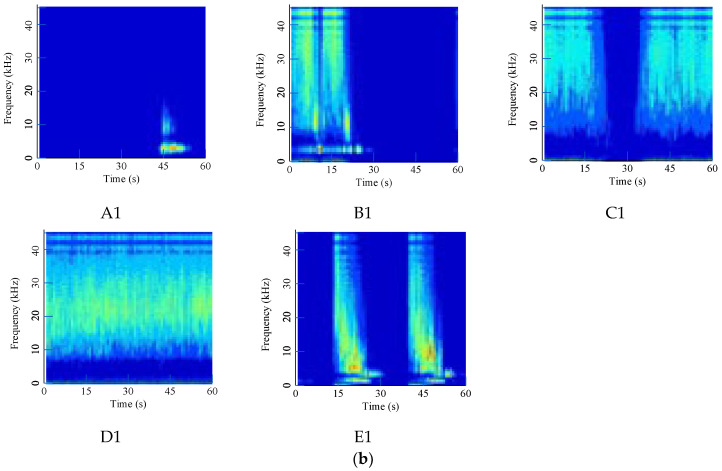
Time-frequency images of denoising acoustic signals: (**a**) Waterwheel room; (**b**) Gallery near the access manhole.

Using the given filtering method, noise is significantly removed, and converted digital images to be diagnosed are shown in Figure 9. The filtered signal has a much clearer shape in the middle and upper area, which represents the high-frequency area. Different faults have special distribution characteristics that can be used for modeling.

### 4.3. Acoustic Diagnosis Model Building

The proposed model is built using the TensorFlow framework in Python 3.9. In addition to the blocks obtained from VGG-16, other layers and the number of parameters are recorded in Table 3. The total number of neurons has decreased to fewer than 100.

The Root Mean Square Propagation (RMSprop) optimization algorithm is used during training, which is commonly used for training neural networks. RMSprop adjusts the learning rates for each parameter individually, which helps in training models with sparse data or noisy gradients. This adaptive learning rate can speed up convergence and prevent oscillations during training. Meanwhile, RMSprop is relatively easy to implement, and it does not require extensive hyperparameter tuning, unlike other optimization algorithms.

**Figure 10:**
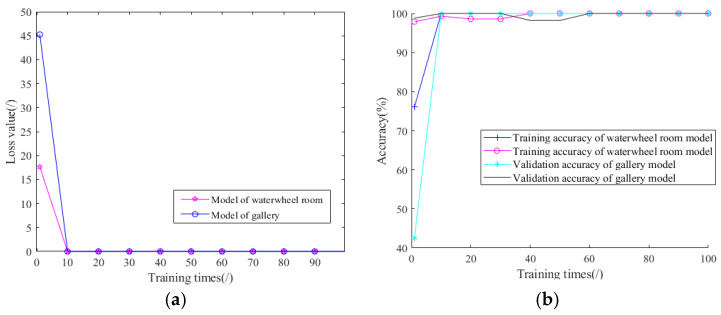
Training curves in waterwheel room: (**a**) Loss value; (**b**) Training accuracy.

Two groups of models are created and trained using a training set and a validation set. The test set is used to evaluate models that did not participate in the training process. In this work, the number of iterations is set to 100 times, and the corresponding loss value and validation accuracy are recorded in Figure 10. Loss values reduce nearly to 0 at about 20 training times. Accuracies increase very quickly. After 30 iterations, the validation training accuracy is 97.62% in the waterwheel room model and nearly 100% for the gallery model. The result reflects that the training process is successful, and the improved model converges quickly.

### 4.4. Results and Discussion

**Figure 11:**
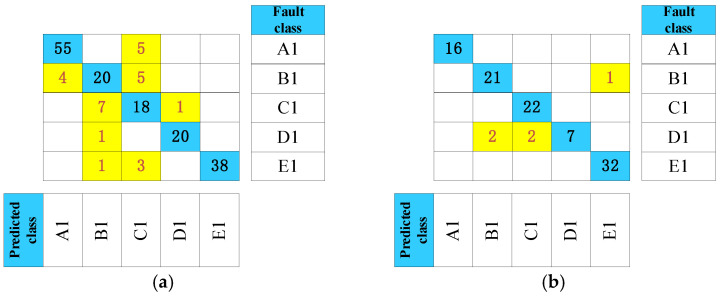
Time-frequency images of original acoustic signals: (**a**) Waterwheel room (**b**) Gallery near the access manhole.

**Figure 12:**
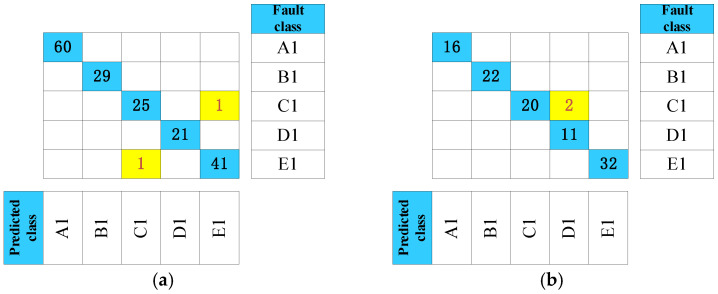
Time-frequency images of denoising acoustic signals: (**a**) Waterwheel room (**b**) Gallery near the access manhole.

After acoustic signal analysis and modeling, a test set is taken to realize an abnormal-sound diagnosis, which includes 178 samples from the waterwheel room and 103 samples from the gallery. To demonstrate the necessity of noise reduction, we added a comparative experiment. The signals before and after filtering were both selected for accuracy comparison. To provide a more specific analysis, confusion matrices are shown in Figure 11 and Figure 12 to evaluate the diagnosis performance of units. The confusion matrix reflects the relationship between the fault class and the predicted class in testing data. Regarding the matrix, values in the row represent the true class, and values in the column mean predicted ones. In the confusion matrices, correctly identified results are marked in blue, while incorrectly identified ones are marked in yellow.

Test data accuracy, precision rate, and recall rate are all used to evaluate the quality of the proposed model, which are commonly used performance metrics in classification problems, as shown in Table 4. The precision rate is the ratio of true positives to the sum of true positives and false positives. Recall rate is used to measure how many of the actual positive instances were correctly classified, which is calculated as the ratio of true positives to the sum of true positives and false negatives.

From Figure 12 and Table 4, test data accuracy reaches 98.88% and 98.06%, respectively. Since abnormal-sound diagnosis is a multiclass classification problem, the precision rate and recall rate should be calculated for each class to obtain a weighted average. Both precision rate and recall values are all over 96% in both groups of results, which means that the proposed model can not only make a diagnosis stably but is not prone to missing fault samples. In general, evaluation metrics reflect that the created model is effective even with limited and unbalanced fault samples from hydroelectric units. Moreover, Class C and Class E may be misjudged as each other due to the similarity between metal rubbing and metal rubbing collision. These two acoustic signals both have a high frequency from metal parts touching.

From Figure 11 and Figure 12, it is evident that the signal accuracy rate before filtering is 84.83% and 95.14%, respectively. After filtering, the accuracy rate increases to 98.88% and 98.06%, demonstrating the effectiveness of the noise-reduction process. In the waterwheel room, the pre-filtering accuracy is considerably lower than the post-filtering results, primarily due to interference from the loud noise and strong power of water flow impacts, which complicates abnormal signal detection. Conversely, the walls in the gallery help block some of the noise, resulting in less pronounced differences compared to the waterwheel room.

### 4.5. Comparative Analysis

To demonstrate the superiority of the proposed model in this work, we have selected a series of classic deep-learning models for comparative analysis, including AlexNet, Resnet18, and MobileNetV3. These methods are widely used in image recognition. Using acoustic signal data after denoising as described in Table 1 and Table 2, comparative models are trained to make a recognition. The accuracy rates are recorded in Table 5.

The overall accuracy for data from the waterwheel room is above 80%. However, after denoising, the testing accuracies for AlexNet and ResNet18 are only 84.27% and 91.01%, respectively, which are insufficient for practical application. MobileNetV3 and VGG16 show better performance yet still fall short compared to the proposed method. Instead of fully training complex models, utilizing pre-trained transfer-learning models combined with a well-designed decision-making module can achieve efficient and accurate recognition results. For data from the gallery near the access manhole, accuracy rates are lower than those from the waterwheel room. This reduction is because the sound signal must pass through the metal walls of the turbine and concrete, significantly attenuating high-frequency components and increasing recognition difficulty. Therefore, the accuracy of the compared models has significantly decreased.

## 5. Conclusions

To realize non-contact measurement in fault diagnosis for a Kaplan hydroelectric generating unit, an abnormal-sound diagnosis method based on CWT and a TL model is proposed. First, this work considers the harshly heavy background noise contained in the water turbine working environment caused by water flow and impaction. A denoising algorithm based on spectral noise-gate technology is provided for acoustic signals in both the waterwheel room and gallery, which can remove heavy noise effectively. Second, a time-frequency analysis based on CWT and visualization is performed to obtain multiple-scales information. All data are compressed and standardized by visualization and then converted into a series of pseudo-color images to highlight information differences and realize recognition. Finally, abnormal-sound signals are collected by an acquisition system with a 96 kHz sampling frequency. To maximize modeling efficiency and minimize modeling costs, an improved TL model based on VGG16 is created to recognize different fault modes by collected data. VGG16 blocks are transferred into the model for image-feature extraction, and simplified fully connected layers are used to eliminate parameters to a large degree.

The experimental results show that the proposed method achieves accuracies of 98.88% and 98.06% in the waterwheel room and gallery, respectively. It also demonstrates high precision and recall rates exceeding 98%, reflecting strong performance even under conditions of imbalanced and small sample sizes. Abnormal-sound diagnosis has a clear advantage of non-contact measurement compared to vibration and swing methods, which are commonly used in unit monitoring. The signal diagnosis accuracy rate before filtering is 84.83% and 95.14%, respectively, and increases to 98.88% and 98.06%, significantly demonstrating the effectiveness of the noise-reduction process. To demonstrate the superiority of the improved model in this work, we have selected a series of classic deep-learning models for comparative analysis.

The proposed method integrates denoising, signal analysis, and transfer-learning techniques for analyzing acoustic signals in hydroelectric units. It effectively addresses various challenges, including acoustic denoising in the waterwheel room, visualization of acoustic signals, and the difficulties associated with learning from imbalanced and small sample sizes. However, while this study primarily focuses on mechanical faults, it does not address certain abnormal operational states of the units that result from changes in the internal flow field. To develop a more comprehensive diagnosis system for hydroelectric generating units based on acoustic signals, future research will elucidate the relationship between abnormal acoustic signals and internal flow field conditions.

## Figures and Tables

**Table 1:** Size and class of acoustic signals in the waterwheel room.

No.	State Type	Training Set (s)	Validation Set (s)	Test Set (s)
Class A1	Normal running	180	60	60
Class B1	Buzzer	87	29	29
Class C1	Metal rubbing	78	26	26
Class D1	Square wave	63	21	21
Class E1	Metal collision	126	42	42

**Table 2:** Size and class of acoustic signals in the gallery near the access manhole.

No.	State Type	Training Set (s)	Validation Set (s)	Test Set (s)
Class A2	Normal running	48	16	16
Class B2	Buzzer	66	22	22
Class C2	Metal rubbing	66	22	22
Class D2	Square wave	33	11	11
Class E2	Metal collision	96	32	32

**Table 3:** Layers and parameters.

Layer	Output Shape	Number of Parameters
Fully connection-1	64	1,605,696
Fully connection-2	32	2080
Output	5	165

**Table 4:** Results of sound diagnosis.

Signal	Position	Test Accuracy (%)	Precision Rate (%)	Recall Rate (%)
Original acoustic signals	Waterwheel room	84.83	83.10	83.12
	Gallery	95.15	95.98	91.82
Denoising acoustic signals	Waterwheel room	98.88	98.75	98.75
	Gallery	98.06	96.92	98.18

**Table 5:** Results of sound diagnosis.

Position	Model	Test Accuracy (%)	Precision Rate (%)	Recall Rate (%)
Waterwheel room	AlexNet	84.27	86.39	84.27
	ResNet18	91.01	89.46	89.12
	MobileNetV3	96.63	96.82	96.63
	TL model	98.88	98.75	98.75
Gallery near the access manhole	AlexNet	61.17	62.04	61.17
	ResNet18	80.58	86.65	80.58
	MobileNetV3	60.19	64.27	60.19
	TL model	98.06	96.92	98.18

## Data Availability

The data presented in this study are available on request from the corresponding author due to the review and approval of the equipment-providing hydropower station.
